# The poplar pangenome provides insights into the evolutionary history of the genus

**DOI:** 10.1038/s42003-019-0474-7

**Published:** 2019-06-18

**Authors:** Bingyu Zhang, Wenxu Zhu, Shu Diao, Xiaojuan Wu, Junqian Lu, ChangJun Ding, Xiaohua Su

**Affiliations:** 1 State Key Laboratory of Tree Genetics and Breeding, Research Institute of Forestry, Chinese Academy of Forestry, 100091 Beijing, China; 2 Key Laboratory of Tree Breeding and Cultivation of State Forestry and Grassland Administration, Research Institute of Forestry, Chinese Academy of Forestry, 100091 Beijing, China

**Keywords:** Plant evolution, Plant sciences

## Abstract

The genus *Populus* comprises a complex amalgam of ancient and modern species that has become a prime model for evolutionary and taxonomic studies. Here we sequenced the genomes of 10 species from five sections of the genus *Populus*, identified 71 million genomic variations, and observed new correlations between the single-nucleotide polymorphism–structural variation (SNP–SV) density and indel–SV density to complement the SNP–indel density correlation reported in mammals. Disease resistance genes (R genes) with heterozygous loss-of-function (LOF) were significantly enriched in the 10 species, which increased the diversity of poplar R genes during evolution. Heterozygous LOF mutations in the self-incompatibility genes were closely related to the self-fertilization of poplar, suggestive of genomic control of self-fertilization in dioecious plants. The phylogenetic genome-wide SNPs tree also showed possible ancient hybridization among species in sections Tacamahaca, Aigeiros, and Leucoides. The pangenome resource also provided information for poplar genetics and breeding.

## Abstract (web summary)

Bingyu Zhang et al. present the pan-genome of Chinese poplars, finding millions of sequence and structural variations and an expansion of disease resistance genes among genes with heterozygous non-functioning variations. They also find evidence of genomic control of self-fertilization in poplar.

## Introduction

Poplar (*Populus* L.) is a genus of trees with economic and ecological importance. Several species (e.g., *Populus trichocarpa*) have become model tree species for genetic, taxonomic, and evolutionary studies due to certain characteristics such as small genome size, fast growth, vegetative propagation, easy transformation, and relatively long lifespan, which exposes them to numerous abiotic and biotic stresses. The existence of many wild individuals (i.e., trees unaffected by artificial selection) and wild species distributed worldwide provides good material for evolutionary studies.

In the traditional classification method, species in the genus *Populus* were classified into six sections based on their morphological traits and crossability: Abaso, Leuce (Populus), Aigeiros, Tacamahaca, Leucoides, and Turanga^1^. With the development of molecular markers and sequencing, their taxonomic relationship was tested by phylogenetic analysis based on DNA markers and DNA sequences. For example, amplified fragment length polymorphism (AFLP) results showed that the genetic distance between *Populus mexicana* Wesmael (section Abaso) and other *Populus* species was larger than that of *Salix* and the genus *Populus*, suggesting that the former belongs to another genus^2^, leaving five sections in the genus *Populus*. Of these five sections, experiments have found the species of three sections (Tacamahaca, Aigeiros, and Leucoides) to be mixed together, unable to form their own sections based on AFLP markers and DNA sequences^3–6^. For example, in one study*, Populus simonii* Carr. in the section Tacamahaca was observed to be excluded from the clade formed by species of the section Tacamahaca^3^; in another study, it formed a cluster with *Populus deltoides* Bartr. and *Populus nigra* L. of the section Aigeiros^6^. Meanwhile, *P. nigra* has shown large discrepancies between traditional and molecular phylogenetic classifications^2,3,6,7^. Such controversial results of the phylogenetic analysis of DNA markers and sequences might stem from differences in the evolutionary rates of the targeted sequences in the poplar genome. Moreover, these molecular phylogenetic studies have indicated that species with distinct morphological characteristics may be genetically closed, suggesting a limitation of traditional taxonomy in this easily outcrossing tree species.

The release of whole-genome data for black cottonwood (*P. trichocarpa* Torr. and Gray)^8^ has enabled the detection of a massive number of genetic variations for the purpose of deciphering the genetic relationship between different species and discovering the evolutionary history of the genus *Populus* at the whole-genome level based on next-generation sequencing. For example, Wang et al.^9^ performed a genome-wide phylogenetic comparative study of three related *Populus* species (*P. tremula* L., *P. tremuloides* Michx., and *P. trichocarpa*) using whole-genome resequencing data and found that natural selection widely shaped the patterns of nucleotide polymorphisms at linked neutral sites in all three species. Meanwhile, genome-wide structural variation (SV) in three intercrossable poplar species (*P. nigra*, *P. deltoides*, and *P. trichocarpa*) have been detected, resulting in the first poplar pan-genome based on genome resequencing data, to our knowledge^10^.

As a natural center of origin of poplars, China has 53 native poplar species belonging to five sections (i.e., no species from section Abaso) of the genus *Populus*, representing over half the *Populus* species worldwide^11,12^. Studies based on the internal transcribed spacer sequence, trnL-F sequence, and AFLP have revealed rich genetic variations in native Chinese poplar species^13–15^. In this work, we resequenced 10 clones from 10 species (i.e., one clone per species) selected from five sections of *Populus* (Supplementary Table 1) with known distribution regions in the wild and species-specific morphological characteristics (referred to as the 10 species hereafter). Except for *P. deltoides* cv. Shanhaiguanensis, all other species were native to China, and nine species (excluding *Populus lasiocarpa* Oliv.) have been used as crossing parents in China^11^. Based on a comprehensive analysis, we discovered substantial DNA variations in the 10 species, performed a phylogenetic analysis, and studied the evolutionary history of these genomic mutants.

## Results

### Sequencing and mapping

Using an Illumina HiSeq 2000 sequencing instrument, we sequenced the 10 poplar species to >33× coverage and yielded 1.81 billion 90-bp paired-end reads, which generated 162.89 G of clean data. The sequencing quality of these clean reads was generally high (>90% with a Phred quality score > 20; > 80% with a Phred quality score >30). The short reads were mapped to the genomic sequence of *P. trichocarpa* v.3.0. Large differences in the mapping rate (lowest: 59.34%, highest: 81.04%) were observed among the 10 sequenced species, where the species in the section Leuce had the lowest mapping rate followed by the species of the section Turanga, and the species of the other three sections had the highest mapping rates (Supplementary Table 2). Studies based on morphological traits and molecular phylogenetic analyses showed that species in the sections Turanga and Leuce were older than those in the sections Tacamahaca, Aigeiros, and Leucoides in *Populus* relative to a species from section Tacamahaca^1,2,5,6^. Therefore, the distinct mapping rate of the 10 species was most likely caused by the genetic divergence of each species from the reference.

### Single-nucleotide polymorphism detection and annotation

Using a strict pipeline, we identified 60.6 million high-quality single-nucleotide polymorphisms (SNPs) in the 10 species. The species in the sections Tacamahaca, Aigeiros, and Leucoides had relatively high numbers of SNPs, whereas those in the sections Leuce and Turanga had relatively few SNPs. Among the identified SNPs, homozygous SNPs were predominant (74.84%) over heterozygous SNPs (25.16%) (Supplementary Table 3). *Populus ussuriensis* Kom. had the highest ratio of heterozygous/homozygous SNPs (0.50), whereas *P. nigra* and *P. euphratica* Oliv. had the lowest (0.19) (Supplementary Table 4). After identifying SNPs located in the genic region, we found that most SNPs (63.66%) were distributed in the intergenic region, and only 9.78% (5,926,381) were located in coding regions (CDS). Among CDS SNPs, over half (53.61%) were caused by non-synonymous changes and could affect the function of 34,495–35,557 genes in each species (Supplementary Fig. 1). The average ratio of non-synonymous to synonymous substitutions (Nonsyn/Syn) of the 10 species was 1.16, with variations among the species (Supplementary Table 5). Species belonging to the same section tended to have similar Nonsyn/Syn ratios. Species of the sections Tacamahaca and Leucoides had the highest Nonsyn/Syn ratios (1.24–1.25), species of the section Aigeiros had medium ratios (1.17–1.18), and species of the sections Leuce and Turanga had the lowest ratios (1.07–1.09).

SNPs were classified as transitions (C/T and A/G) or transversions (A/T, A/C, T/G, and C/G) based on nucleotide substitution. There were more transitions than transversions, and the transition/transversion ratio of the 10 species ranged from 1.39 to 1.86 (Supplementary Table 6). In the 10 species, there were similar numbers of C/T transitions and A/G transitions but relatively more A/T transversions than other transversions.

About 39,164 SNPs (~0.06% of all SNPs) were shared among the 10 species, and all of the common SNPs were homozygous (Supplementary Data 1). Gene Ontology (GO) enrichment analysis of the genes with common SNPs was performed using agriGO^16^. Fourteen GO terms related to regulation of transcription, metabolic processes, and biosynthetic processes were significantly enriched (corrected *p* < 0.05, FDR < 0.05) (Supplementary Table 7).

### Identification of large-effect SNPs

SNPs predicted to have a potential disabling effect on gene function were considered large-effect SNPs^17^. We identified 155,466 large-effect SNPs (0.26% of the total SNPs) in the 10 species, including 103,752 SNPs (66.74% of the large-effect SNPs) that turned start codons into non-start codons, 42,856 SNPs (27.57%) causing premature stops, and 12,242 SNPs (5.70%) that turned stop codons into non-stop codons (Supplementary Fig. 2, Supplementary Table 8).The number of large-effect SNPs varied among the 10 species, and species with more total SNPs usually had fewer large-effect SNPs. For example, *Populus alba* L., *Populus davidiana* Dode., and *P. euphratica* had fewer total SNPs but more large-effect SNPs. The differences in the number of large-effect SNPs among the 10 species may have stemmed from the use of *P. trichocarpa* as reference, which had a larger genetic distance from *P*. *alba, P. davidiana*, and *P. euphratica* than from the other species.

Gene Ontology enrichment analysis of genes with the three types of large-effect SNPs in each species was performed using agriGO. Significantly enriched GO terms (corrected *p* < 0.05, FDR < 0.05) were found in genes with two types of large-effect SNPs (non-start codon SNPs and stop codon SNPs). About 22–57 significantly enriched GO terms were found in genes with non-start codon SNPs of the 10 species (Supplementary Data 2). The GO terms death, cell death, programmed cell death, and apoptosis death were found in all species except *P. euphratica*; the GO terms cell recognition, reproduction, pollination, pollen–pistil interaction, reproductive process, and recognition of pollen were found in two species (*P. alba* and *P. davidiana*) of the section Leuce, two species (*Populus cathayana* Rehd. and *P. simonii*) of the section Tacamahaca, and *P. deltoides* of the section Aigeiros. The cellular component GO terms (e.g., external encapsulating structure part, periplasmic space, and cell envelope) and molecular function GO terms (e.g., ligand-gated channel activity and ligand-gated ion channel activity) were found in only *P. cathayana*. About 22–40 significantly enriched GO terms were found in genes with stop codon SNPs for the 10 species (Supplementary Data 3); the GO terms death, cell death, programmed cell death, apoptosis death, protein kinase activity, and transferase activity were significantly enriched in all 10 species; the GO terms immune system process, innate immune response, and immune response were found in only five species (*P. davidiana*, *P. simonii*, *P. ussuriensis*, *P. maximowiczii* Henry, and *P. lasiocarpa*); and the reproduction GO terms (e.g., cell recognition, reproduction, pollination, pollen–pistil interaction, reproductive process, and recognition of pollen) were found in only *P. cathayana* and *P. ussuriensis*. The differences in the significantly enriched GO terms of the genes with large-effect SNPs in these poplar species gave clues regarding natural selection on certain genes during their long evolutionary process.

### Small insertion and deletion detection and annotation

We detected 10,317,787 small insertions and deletions (indels;<6 bp); *P. euphratica*, *P. alba*, *P. davidiana*, and *P. lasiocarpa* had fewer indels (<100,000), whereas the other species had more (>100,000). Among the identified indels, there were 5,113,532 insertions and 5,204,255 deletions, and about 19,458 common indels (~0.22% of the total) were shared among the 10 species (Supplementary Data 4). Indels located in the genic region were identified; about 7.82% were located in untranslated regions, and 2.52% were in CDS (Supplementary Table 9). Compared to SNPs, the percentage of indels in CDS was much lower, likely because indels in CDS have greater effects than SNPs on gene function through their influence on transcripts and protein structures; therefore, they have been selected against during evolution, as described previously^18^. Of the indels located in CDS, over half caused frameshift mutations (58.94%), resulting in changes in 7,125–9,992 protein coding genes in the 10 species (Supplementary Fig. 3).

GO term enrichment analysis of the frameshift genes in each species was performed with agriGO, from which 11–36 significantly enriched GO terms (corrected *p*-value < 0.05, FDR < 0.05) were identified in the 10 species. The GO terms cell death, programmed cell death, apoptosis, and death were significantly enriched in all 10 species (Supplementary Data 5). Species-specific significantly enriched GO terms were found only in two species: *P. davidiana* had seven enriched GO terms in biological process (macromolecule modification, reproduction, pollination, cell recognition, reproductive process, recognition of pollen, and pollen–pistil interaction) and one in molecular function (sugar binding); and *P. maximowiczii* had two in molecular function (heme binding and iron ion binding). Additionally, most of the significantly enriched programmed cell death genes coded for R proteins. Indel markers have been used in genetic mapping of plants, and indels significantly correlated with disease have also been identified in the peanut^19–21^. The indels identified in each species could provide genome-wide candidate molecular markers for disease-resistance breeding and genetic studies of *Populus*.

### Structural variation detection and annotation

In total, 200,501 SVs were identified, among which deletions were much more common than insertions and other types of SVs. The deletion/insertion ratio differed among the 10 species; *P. davidiana* had the highest ratio (9.35), *P. euphratica* had the lowest ratio (3.70), and *P. lasiocarpa* had no insertions (Table 1). Because the mapping rate of *P. lasiocarpa* was typical of the 10 species (Supplementary Fig. 4), the difference might not come from variation in mapping efficiency. This unusual phenomenon should be confirmed in future studies. Among the SVs identified, nearly half (46.05%) were located in CDS, 65.02% of which were deletions and 13.07% of which were insertions, suggesting that poplar genes are more tolerant of large deletions than of large insertions (Supplementary Fig. 5). Expression of the genes affected by SV was significantly reduced in poplars^10^. The high-frequency distribution of SVs in genic regions of all 10 poplar species suggested the involvement of SVs in gene regulation in poplar.

**Table 1:** Summary of all the detected SVs of the 10 poplar species

Sections/speices	SV	INS	DEL	ITX	CXT	INV	D/I
Sect. Leuce Duby							
* P. alba* L.	14,850	1555	11,309	481	1326	179	7.27
* P. devidiana* Dode.	16,710	1378	12,891	571	1644	226	9.35
Sect. Tacamahaca Spach.							
* P. cathayana* Rehd.	24,619	2585	16,775	1,161	3602	496	6.49
* P. simonii* Carr.	23,119	3094	15,002	882	3708	433	4.85
* P. ussuriensis* Kom.	26,393	3403	17,403	1253	3794	540	5.11
* P. maximowiczii* Henry.	27,183	3,940	17,792	1182	3761	508	4.52
Sect. Aigeiros Duby.							
* P. nigra* L.	20,594	2876	13,700	769	2870	379	4.76
* P. deltoides* Bartr.	18,973	1522	13,776	786	2504	385	9.15
Sect. Leucoides Spach.							
* P. lasiocarpa* Oliv.	19,063	0	13,642	1,024	3910	487	
Sect. Turanga Bge.							
* P. euphratica* Oliv.	8997	1674	6195	225	779	124	3.70
Total	200,501	22,027	138,485	8334	27,898	3757	
Percentage of the total		10.99	69.07	4.16	13.91	1.87	

*D/I* deletions/insertions, *DEL* deletion, *INS* insertion, *INV* inversion, *ITX* translocation

The largest deletions in each species were about 18–50 Mbp, and the corresponding regions in the reference gene contained 1526–4573 genes (Supplementary Fig. 6). The largest deletions in each species were located in the longest chromosome (chromosome 01). Clusters of genes (ankyrin repeat genes, LRR protein genes, NBS-LRR disease resistance genes, NB-ARC disease resistance genes, cysteine-rich receptor-like protein kinase genes, and heavy metal transport/detoxification superfamily genes) were found in large deletions. We performed GO enrichment analysis of the genes located in one of the large deletions of the 10 species and found that resistance-associated gene families were significantly enriched (Supplementary Fig. 7), similar to the findings of studies of other plant species^22–24^.

### Phylogenetic relationship among the 10 Populus species

Using the SNPs identified in the 10 species, phylogenetic trees based on SNPs were constructed to infer their relationship at the whole-genome level.

**Fig. 1:**
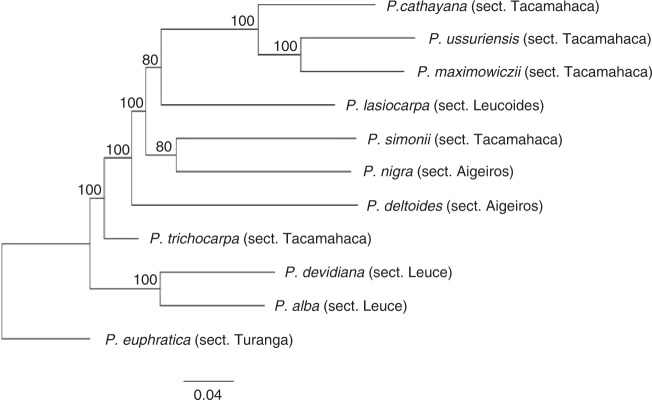
Phylogenetic tree constructed based on the maximum likelihood method using whole-genome SNPs identified in 10 poplar species, showing the relative genetic relationship between the 10 species from five sections of the genus *Populus*. The tree was imaged with FigTree (ver. 1.4.3)

The phylogenetic tree contained three distinct branches: one branch of *P. euphratica* (section Turanga), one branch formed by *P. alba* and *P. davidiana* (section Leuce), and species from the sections Tacamahaca, Aigeiros, and Leucoides grouped as one branch (Fig. 1). *P. simonii* and *P. nigra* were clustered together, and the three species of the section Tacamahaca (*P. maximowiczii*, *P. ussuriensis*, and *P. cathayana*) were closely grouped. In addition, *P. lasiocarpa* was grouped with the cluster formed of three species of the section Tacamahaca. *P. deltoides* and the reference *P. trichocarpa* were grouped with the cluster formed by species from the sections Tacamahaca, Aigeiros, and Leucoides.

Based on the whole-genome SNP trees, we found that species of the sections Turanga and Leuce could be distinguished from species of the other three sections (Tacamahaca, Aigeiros, and Leucoides), but species within Tacamahaca, Aigeiros, and Leucoides were clustered and mixed together, indicating that they were genetically closely related. In contrast to traditional classification, the phylogenetic trees revealed several distinctions in the relationships of the species; species of the section Tacamahaca, Aigeiros, and Leucoides could not be distinguished; two species of the section Aigeiros were not clustered together; and *P. simonii* was not grouped with other species of the section Tacamahaca.

### Genome-wide correlations among SNP, indel, and SV density

**Fig. 2:**
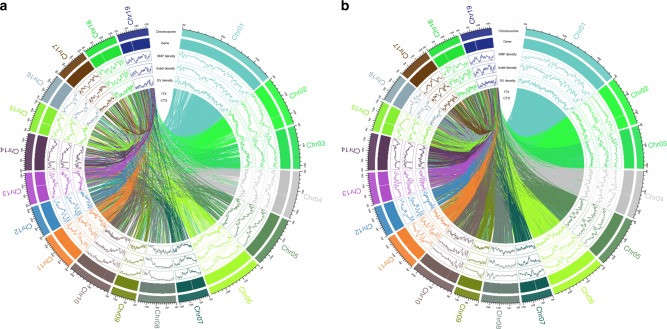
Circos plot presenting the distribution of SNPs, indels, and SVs on 19 poplar chromosomes in *P. alba* (**a**) and *P. cathayana* (**b**): outer, chromosome, gene density, SNP density, indel density, SV density, and ITX and CTX. The scales are indicated on the outer rim. The density is defined as the number of SNPs/indels/SVs per 500 Kb (excluding CTXs and ITXs from the SV density calculation). CTXs and ITXs were also presented in the middle of the circus plot. The Chromosome distributions of the genomic variation of other species are not presented

An uneven chromosome distribution of SNPs, indels, and SVs was observed in all 10 species, and regions with high densities of these variations tended to be located at the same genomic region (Fig. 2). We explored the correlations among the densities of SNPs, indels, and SVs (excluding interchromosomal (CTXs) and intrachromosomal (ITXs) translocations) (bin: 500 kb, step: 50 kb). The results showed that the SNP, indel, and SV densities in all species were significantly correlated with each other, but the correlation coefficients varied (Table 2, Supplementary Figs. 8 and 9). The correlation coefficients of the SNP–indel density, indel–SV density, and SNP–SV density in the species of the two sections Turanga and Leuce were higher than those of the other three sections (Tacamahaca, Aigeiros, and Leucoides). Moreover, the correlation coefficients of the SNP–SV density in species of the sections Tacamahaca, Aigeiros, and Leucoides were much lower than those of the SNP–indel density and the indel–SV density in the same species.

**Table 2:** Pearson correlations between SNP, indel, and SV densities in the genomes of the 10 poplar species

	1	2	3	4	5	6	7	8	9	10
SNP × indel density	0.97	0.96	0.63	0.70	0.67	0.68	0.75	0.76	0.71	0.99
SNP × SV density	0.84	0.84	0.4	0.46	0.39	0.45	0.49	0.45	0.40	0.82
Indel × SV density	0.87	0.88	0.69	0.76	0.66	0.70	0.78	0.73	0.61	0.83

Notes: a: 1: *P.alba;* 2: *P. devidiana*; 3: *P.cathayana*; 4: *P. simonii*; 5: *P. ussuriensis*; 6: *P. maximowiczii*; 7: *P. nigra*; 8: *P. deltoids*; 9: *P. lasiocarpa*; 10: *P. euphratica*

b: The density was defined to be the number of SNPs/indels per 500 Kb, and all the *P*-values were near to zero (*P*≈0)

### PCR validation of SNPs, indels, and SVs

To validate some of the putative SNPs and indels identified by next-generation resequencing, we amplified and sequenced 20 small fragments (383–1021 bp) from the genome DNA of two species (*P. alba* and *P. deltoides*) used for resequencing (Supplementary Tables 10 and 11). Among the 270 putative SNPs predicted by bioinformatics, 257 were validated, with a total validation rate of 95.19%. Among the 67 putative indels, 58 were validated, with a total validation rate of 86.57% (Table 3). A few additional SNPs and indels were also detected. The total validation rate of SNPs was higher than that of indels. Overall, the validation results showed that the sequencing and variation calls were generally reliable.

**Table 3:** Validation results of SNPs and indels of two species (*P. alba* and *P.deltoides*) by PCR and Sanger sequencing

Variation	Species	Variation predicted	Validation				Validation rate (%)^a^	FP rate (%)^b^	FN rate (%)^c^
			PCR	True	FP	FN			
SNPs	*P. alba*	186	177	173	13	4	93.01	6.99	2.15
	*P.deltoides*	84	87	84	0	3	100	0	3.57
	Total	270	264	257	13	7	95.19	4.81	2.59
Indels	*P. alba*	46	39	37	9	2	80.43	19.57	4.35
	*P.deltoides*	21	23	21	0	2	100	0	9.52
	Total	67	62	58	9	4	86.57	13.43	5.97

^a^*FP* false positive, *FP rate * FP variations/variations detected by software × 100%

^b^*FN* false negative, *FN rate*  FP variations/variations detected by software × 100%

^c^*Validation rate* True variations/variations detected by software × 10

Six predicted deletions of 2.2–2.6 kb in the genome of *P. lasiocarpa* were randomly selected from the SV dataset, and primers within the deletion region were designed according to the corresponding *P. trichocarpa* sequence (Supplementary Table 12). These primers obtained the predicted sizes of PCR products from *P. trichocarpa* genomic DNA but did not yield products from the *P. lasiocarpa* DNA (Supplementary Fig. 10). The validation results suggested that the deletions predicted in this study were generally accurate.

## Discussion

Large-effect SNPs have been reported in plants, such as rice, maize, and sorghum^17,22,25,26^. In this study, large-effect SNPs were found in all 10 species of the five sections of *Populus*, indicating the ubiquity of large-effect SNPs in this genus. However, only a small proportion (0.18–0.41%) of SNPs in each species were identified as large-effect SNPs, indicative of the detrimental function of these SNPs, which is similar to those in mammalian genomes^18,27^.

Studies of annual herbs and perennial woody plants showed that disease resistance genes (R genes) were usually significantly enriched in the genes with large-effect SNPs^17,28,29^. In our study, R genes (NB-ARC domain-containing disease resistance genes, LRR domain-containing disease resistance genes, and TIR/CC-NBS-LRR class disease resistance genes) involved in programmed cell death, cell death, apoptosis, and death were significantly enriched in genes with large-effect SNPs in all 10 poplar species. Such R genes were also significantly enriched in genes with frameshift indels, and clustered in large deletions in these poplar species. Further GO enrichment analyses of the genes with homozygous and heterozygous large-effect SNPs found that genes with heterozygous large-effect SNPs were significantly enriched in R genes of all 10 poplar species (Supplementary Data 6–8). Similarly, genes with heterozygous frameshift indels were significantly enriched in the R genes of these poplar species (Supplementary Data 9 and 10). Generally, the effect of a heterozygous mutation in diploids is expected to be reduced or completely sheltered by the original allele unless it is fully dominant. Even in the case of full dominance, the lost resistance caused by the LOF mutations in R genes might be compensated for by other members of R gene families. Due to the dioecious nature of the genus *Populus*, these heterozygous LOF mutations had little chance to become homozygous and were kept in their genomes. In addition, the long battle between pathogens and poplars enabled the evolution of more R genes in their genomes; for example, over 400 NBS-LRR genes have been found in the genome of *P. trichocarpa*^30^. These newborn genes further increased R gene polymorphism, together with heterozygous loss-of-function R genes and ancestral R genes, and protected poplars from infection by multiple pathogen species, ultimately increasing their fitness.

Furthermore, we found that reproduction-related GO terms (e.g., cell recognition, pollination, pollen–pistil interaction, reproductive process, and recognition of pollen) were significantly enriched in genes with large-effect SNPs in 6 of the 10 species (*P. alba*, *P. davidiana*, *P. cathayana*, *P. simonii*, *P. ussuriensis*, and *P. deltoides*) and in genes with frameshift indels in *P. davidiana*. The genes involved in reproduction encoded for S-locus lectin protein kinase family proteins, receptor protein kinases, receptor-like protein kinases, and lectin protein kinase family proteins (S-locus proteins hereafter), and about 38.21% of them were homologs of S-locus cysteine-rich protein (SCR) in *Brassicaceae* (Supplementary Data 11). The SCR gene encoded proteins as male determinants of recognition specificity and controlled self-incompatibility (SI) along with the S-locus receptor kinase (SRK)^31–33^. The large-effect SNPs and frameshift indels should cause the S-locus protein to lose its function and reduce the SI of the host. The fixation of a nonfunctional allele (ψ*SCR1*) at the SCR loci was believed to underlie the transition of *A. thaliana* from ancestral SI to full self-compatibility^34^. Then, these loss-of-function S-locus proteins might cause self-fertilization in poplar. There are few reports of self-fertilization in poplar, because species in the genus *Populus* are generally dioecious. However, very few andromonoecious and gynomonoecious individuals have been found in natural populations of *Populus tomentosa* (Sect. Leuce)^35,36^. Bisexual flowers exhibiting self-fertilization were also reported in *P. deltoids*^37^. We found bisexual phenomena in a hybrid of *P. alba* and *P. davidiana*, and in a clone from a natural population of *P. simonii*, e.g., male and female inflorescences on the same tree, or male and female flowers in the same inflorescence, and seeds were obtained by self-pollination (unpublished). As four (*P. alba*, *P. davidiana*, *P. simonii*, and *P. deltoides*) of the six species in our study have bisexual flowers and self-compatibility, we suspect that the large-effect SNPs and frameshift indels located in these self-incompatibility-related genes contributed to the self-fertilization of these poplar species, and self-fertilization clones were expected in populations of the other two species. GO enrichment of genes with homozygous and heterozygous large-effect SNPs revealed that the reproduction-related GO terms were significantly enriched only in genes with heterozygous large-effect SNPs and heterozygous frameshift indels (Supplementary Data 6, 7, and 9). We postulate that these large-effect SNPs or frameshift indels in several S-locus genes occasionally become homozygous and cause bisexual flowers and self-fertilization in a few individuals of these species. However, the rate of simultaneous mutations in several genes was low. In addition, self-incompatibility of dioecious plants is important for maintaining heterozygosity, and self-fertilization may cause inbreeding depression due to deleterious recessive alleles. So a self-fertilized poplar clone may have little chance to have progeny and survive. These might be the main reasons that bisexual clones have rarely been found in populations of these species. However, in-depth functional studies of the LOF variations in the S-locus of poplar are needed to test this hypothesis.

Correlations between SNP and indel density have been reported in mammalian genomes^18,38^. Meanwhile, in plants, markedly elevated nucleotide diversity has been observed near indels and SVs in the cucumber genome^39^. Therefore, we performed a correlation analysis between SNP and SV density, as well as indels and SV density. Two types of SVs (CTXs and ITXs) were not included in the density calculation because these SVs are linked to two chromosome locations. In addition to a SNP–indel density correlation, SNP–SV density and indel–SV density correlations also existed in poplars. Covariations in the frequencies of substitutions, deletions, transpositions, and recombinations have been observed in the human genome^40^. In addition, it has been proposed that the heterozygosity of indels is generally mutagenic to surrounding sequences in primates, rodents, fruit flies, rice, and yeast^41^. Given these results, we speculate that the covariation between SV density and the densities of SNPs and indels might be a common genomic feature in eukaryotes. In the genomes of eukaryotes, some chromosome regions are hot spots for genomic variations, which are assumed to underlie the phenotype selected during species evolution.

Based mainly on morphological characteristics, species in the genus *Populus* have been traditionally classified into five sections^1,11^. However, in subsequent molecular phylogenetic analyses based on DNA sequences, species of the sections Tacamahaca, Aigeiros, and Leucoides have always been mixed together^2–6^. Similarly, in this study, the whole-genome SNP tree could separate these three sections. For example, *P. simonii* has traditionally been a species in the section Tacamahaca. However, in molecular phylogenetic analyses, it has been grouped with species of the section Tacamahaca or has formed a clade with *P. deltoides* and *P. nigra* of the section Aigeiros^6^. In this study, it was separated from the section Tacamahaca and grouped with *P. nigra*, indicating that *P. simonii* is genetically closer to species in the section Aigeiros than to those in the section Tacamahaca at the whole-genome level. In traditional classification, *P. nigra* is classified in the section Aigeiros; however, based on chloroplast sequences, *P. nigra* showed a close affinity to species of the section Populus (i.e., Leuce)^3,6,7^. By contrast, based on its nuclear DNA sequence, some studies have shown a close relationship to the species of the section Aigeiros, whereas others have shown it as distinct from species of the section Aigeiros^2,3,7^. Rajora and Dancik^42^ even proposed a new section, ‘Nigrae,’ containing only *P. nigra*. In this study, the whole-genome sequencing data showed that *P. nigra* was genetically closer to species of the section Tacamahaca than to *P. deltoides* of the section Aigeiros. Together with previous molecular phylogenetic results, these findings suggest that hybridization or gene flow might have occurred in the ancestor(s) of the species of the sections Tacamahaca, Aigeiros, and Leucoides, and traditional classification methods based mainly on morphological characteristics have some limitations in distinguishing these closely related sections. Due to the genetic divergence of species in the genus *Populus*, the genome coverage rate of the 10 species was low, especially in 3 species (*P. euphratica*, 54.16%; *P. alba*, 58.90%; *P. davidiana*, 59.05) (Supplementary Table 2). This means that we lost the genetic information for these uncovered regions. We consider this another reason for the mixture of species of the sections Tacamahaca, Aigeiros, and Leucoides in our phylogenetic SNP tree. Large-scale sequencing has brought new insights into traditional classification, and a few species have had their classifications changed based on de novo sequencing^43^. We propose that the taxonomic classification of the genus *Populus* be renewed based on genomic de novo sequencing data.

Of the five original sections, species in the sections Tacamahaca, Aigeiros, and Leucoides are thought to be modern poplars, whereas species in the sections Turanga and Leuce are thought to be older^1,2,5,6^. However, the oldest species remain disputed; some researchers have suggested that species in the section Leuce are the oldest^2,5^, whereas others have shown species in the section Turanga to be the oldest^1,6^. Synonymous substitutions (Ks) are neutral mutations that can be used to estimate the time since divergence of two species^44^. Using the whole-genome sequencing data, we calculated the Ks values of the 10 species. The results showed that species in the section Turanga had the highest Ks values, species in the section Leuce had moderate Ks values, and species in the other three sections had the lowest values (Supplementary Table 13). Based on the whole-genome sequencing results, we believe that species in the section Turanga are the oldest poplars.

In summary, we performed a genome-wide comparative analysis of 10 *Populus* species belonging to the five original sections, provided the first detailed description of the genomic variation of the genus *Populus* across the whole genome, to our knowledge, found genome-wide significant correlations between three genomic variations (SNP, indel, and SV density), and identified differences from traditional taxonomy in their molecular phylogenetic relationships. We also found that R genes with heterozygous large-effect SNPs and those with heterozygous frameshift indels were enriched in the genomes of the 10 poplar species, and that R genes were very dynamic during the evolution of *Populus*. Finally, the heterozygous large-effect SNPs and frameshift indels located in self-incompatibility-related genes might contribute to self-fertilization in 6 poplar species.

## Methods

### Sample preparation

The 10 species were collected from their natural distribution areas (Supplementary Table 1) and planted in a greenhouse in Beijing, China. DNA was extracted from leaves using the DNeasy Plant Mini Kit (QIAGEN, Hilden, Germany) and quantified on a NanoDrop spectrophotometer (Thermo Scientific, Waltham, MA, USA).

### Library construction and sequencing

Approximately 3.0 μg of total DNA was used for library construction by the Illumina TruSeq DNA Sample Preparation kit (Illumina Inc., San Diego,CA, USA) according to the manufacturer’s instructions. The library was quantified using a DNA 1000 chip on an Agilent Bio-Analyzer 2100 (Agilent Technologies, Palo Alto, CA), and using a standard TaqMan PCR kit protocol on an Applied Biosystems StepOne Plus Real-TimePCR System (Applied Biosystems, Foster City, CA). The genomic DNA library was sequenced on the HiSeq 2000 System (Illumina, San Diego, CA, USA) at the Beijing Genomics Institute (Shenzhen, China).

### Reads mapping and DNA polymorphism calling

After removing sequencing adapters and PCR amplification reads by an in-house Perl script, the clean reads from the sequencing libraries were obtained and mapped to the reference genome. We used *P. trichocarpa* genome v.3.0 (ftp://ftp.jgi-psf.org/pub/compgen/phytozome/v9.0/Ptrichocarpa/) as the reference for variation calling and annotation. The reads of the 10 species were mapped against the *P. trichocarpa* genome using BWA ver. 0.6.1-r104 (settings: mem -t 4 -k 32 –M -R), with five mismatches^45^. Alignment files were converted to BAM files using SAMtools software^46^ (settings: –bS –t). In addition, potential PCR duplications were removed using the SAMtools command rmdup. If multiple read pairs had identical external coordinates, only the pair with the highest mapping quality was retained. SNP and small indel detection were performed with HaplotypeCaller in GATK (v2.5-2) using the default options and filtered using the VariantFiltration tool in GATK with the parameters QD < 20.0 || ReadPosRankSum < −8.0 || FS > 10.0 || QUAL > 20 || DP > 5 || MQ > 30^47^. To reduce the SNP detection error rate, we filtered the SNPs with fewer than five supported reads or quality values less than 30. Small indels were filtered in a similar manner to SNPs, removing those near other variants and within the PAR. SnpEff (ver. 3.2) was used for the functional annotation of the variations.

Structural variations (SVs) such as insertions, deletions, inversions, and intrachromosomal (ITXs) and interchromosomal (CTXs) translocations, was detected using BreakDancer^48^. SVs with fewer than five supported PE reads were filtered out.

The protein sequences of SNP-impacted genes were extracted and aligned with ClustalW (ver. 2.1). Next, the Ks values of SNP-impacted genes were calculated with KaKs Calculator based on the GY-HKY model.

### Phylogenetic analysis

Whole-genome SNPs were used to calculate the genetic distances among species. The SNP site sequences of each sample were extracted as a FASTA file, and sequence multi-alignment was performed with ClustalW (ver. 2.1)^49^, and RaxML was used to reconstruct the phylogenic tree based on the GTR model^50^. Finally, FigTree (ver. 1.4.3) was used to display the tree.

### Correlation analysis among SNP, indel, and SV density

The densities of SNPs, indels, and SVs (excluding CTXs and ITXs) were calculated as the number of each variation event per base pair across the whole genome (bin: 500 kb, step: 50 kb). Pearson correlation coefficients among SNP, indel, and SV density were computed using the *pcor* function in R (http://www.r-project.org/).

### Gene ontology (GO) analysis

GO analysis in this study was performed using agriGO (http://bioinfo.cau.edu.cn/agriGO/) and *p-*values were corrected using the FDR function in R.

### Validation of SNPs, indels, and SVs by Sanger sequencing

We randomly selected 20 short fragments in two species (*P. alba* and *P. deltoids*, randomly selected from the 10 species) for SNP and indel validation by PCR and Sanger sequencing (Supplementary Tables 10 and 11). A standard PCR protocol was executed using HotStar Taq DNA Polymerase (QIAGEN). Then, 5-μL aliquots of the PCR products were run on 1.4% agarose gels and stained with ethidium bromide, and 45-μL aliquots were further purified and sequenced using a 3730xl DNA analyzer (Applied Biosystems, Foster City, CA, USA). Finally, deletions of 2.2–2.6 kb in *P. lasiocarpa* were experimentally validated by PCR amplification (Supplementary Table 12).

### Statistics and reproducibility

Pearson correlation coefficients among SNP, indel, and SV density were computed using the *pcor* function in R. The *p-*values of GO analysis were corrected using the FDR function in R.

### Reporting summary

Further information on research design is available in the Nature Research Reporting Summary linked to this article.

## Supplementary information

Supplementary information

Description of Additional Supplementary Files

Supplementary Data 1

Supplementary Data 2

Supplementary Data 3

Supplementary Data 4

Supplementary Data 5

Supplementary Data 6

Supplementary Data 7

Supplementary Data 8

Supplementary Data 9

Supplementary Data 10

Supplementary Data 11

Reporting Summary

## Data Availability

The data of this study is available in SRA (PRJNA540895).
