# Supplementary material for: The poplar pangenome provides insights into the evolutionary history of the genus
Source: Commun Biol. 2019 Jun 18;2:215. doi: 10.1038/s42003-019-0474-7 (PMC6581948; doi:10.1038/s42003-019-0474-7)
Supplement: Supplementary file 1 — Supplementary information [file 42003_2019_474_MOESM1_ESM.pdf]

Supplementary Figures

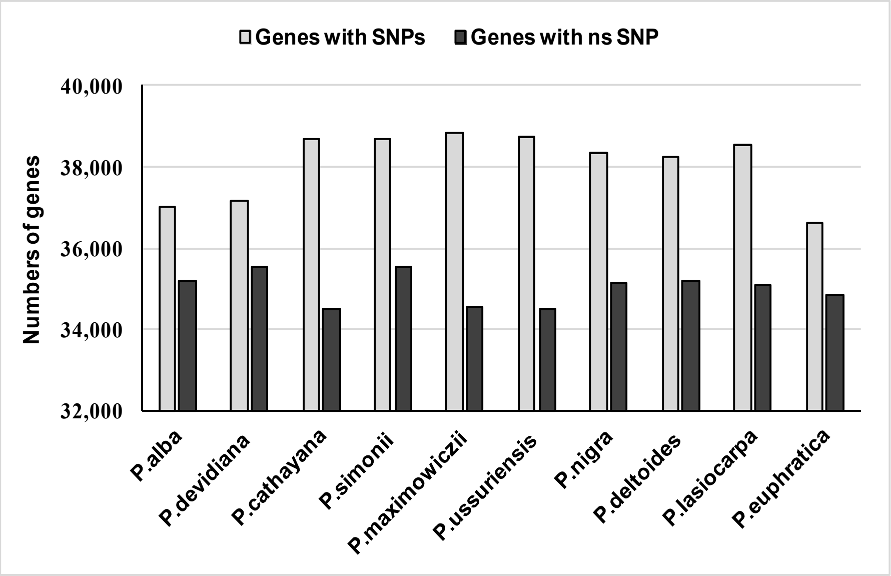

Supplementary Figure 1 Comparison of the numbers (shown by bar lengths) of genes containing SNPs and non-synonymous SNPs (nsSNPs) in the 10 poplar species.

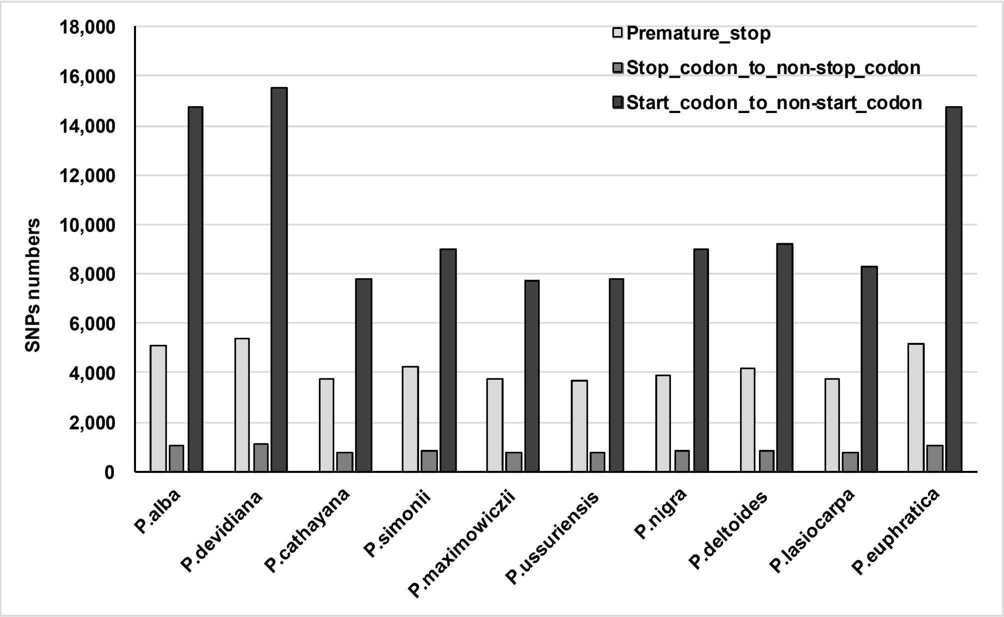

Supplementary Figure 2 Number of various types of large-effect SNPs in the 10 poplar species.

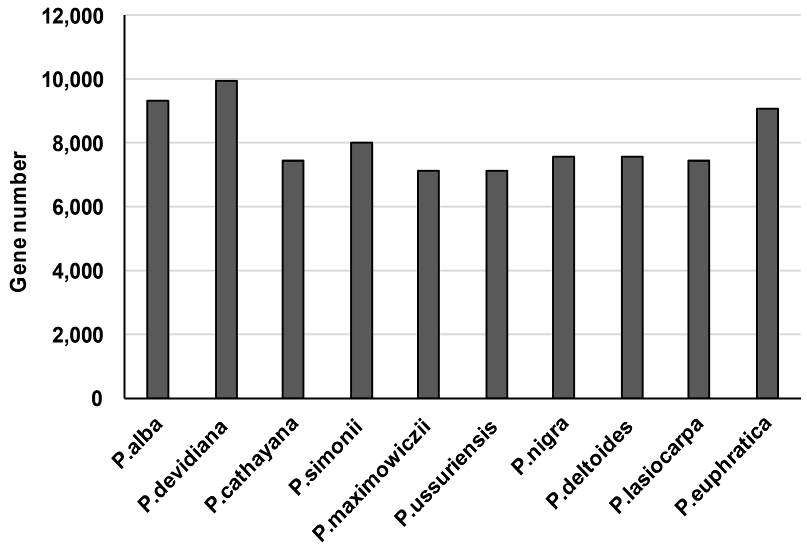

Supplementary Figure 3 Number of genes impacted by frameshift indels in the 10 poplar species.

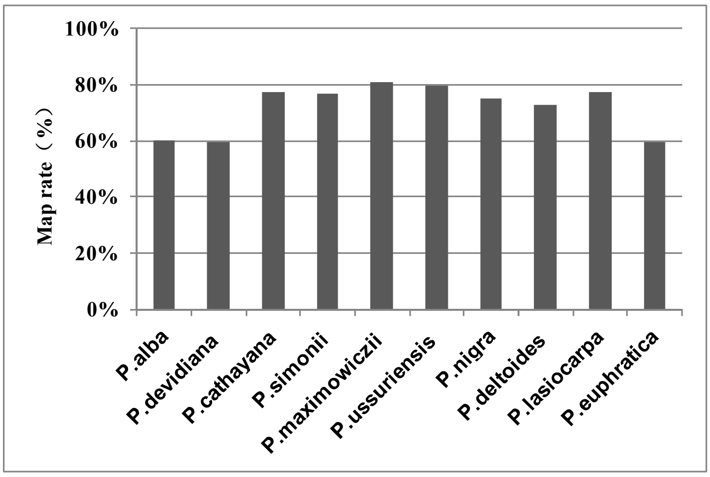

Supplementary Figure 4 Mapping rate of the 10 poplar species.

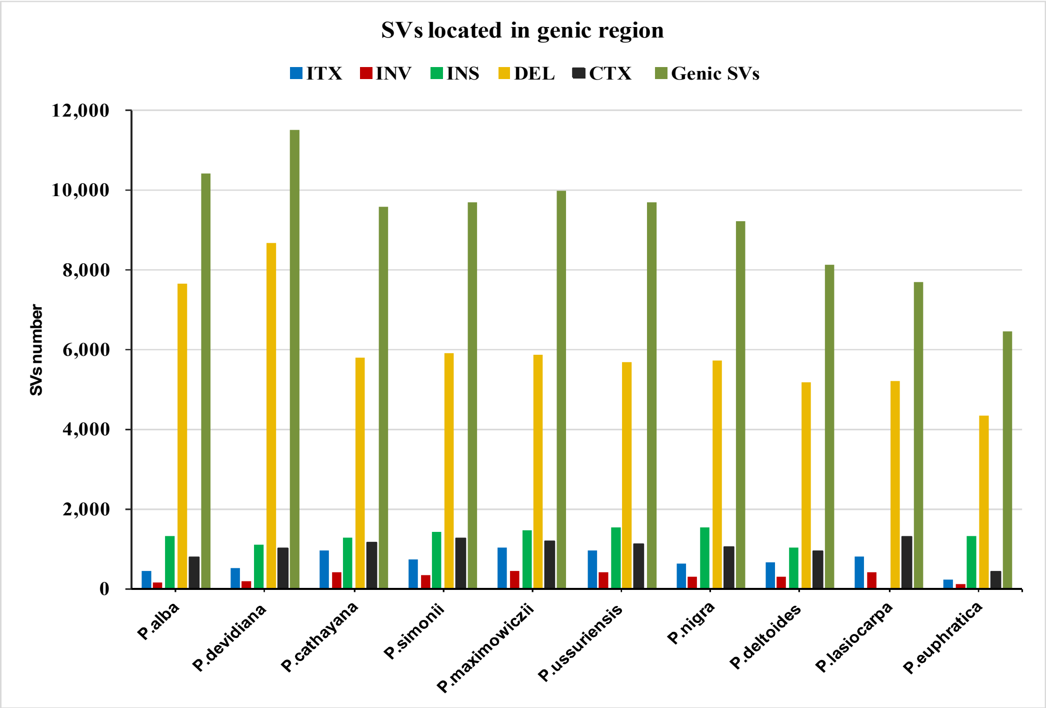

Supplementary Figure 5 SVs in the genic regions of the 10 poplar species. Most of the genic SVs were deletions (65.02%), although 13.07% were insertions, 11.06% were CTXs, 7.57% were ITXs, and 3.29% were INV, indicating that poplar genes are more tolerant of large deletions than other types of SVs.

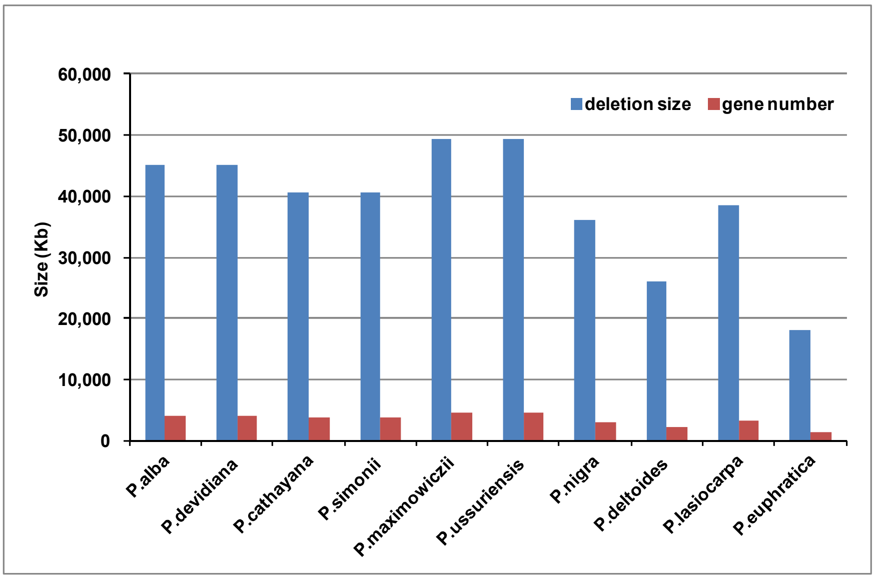

Supplementary Figure 6 Size of the largest deletions in the 10 poplar species and the number of genes located in these largest deletions in the corresponding regions of the reference genome.

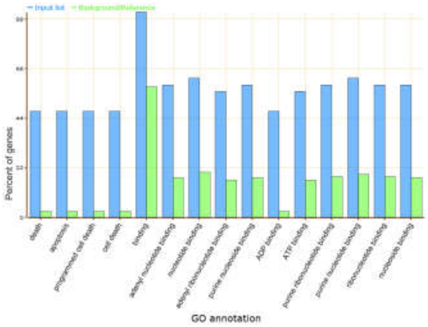

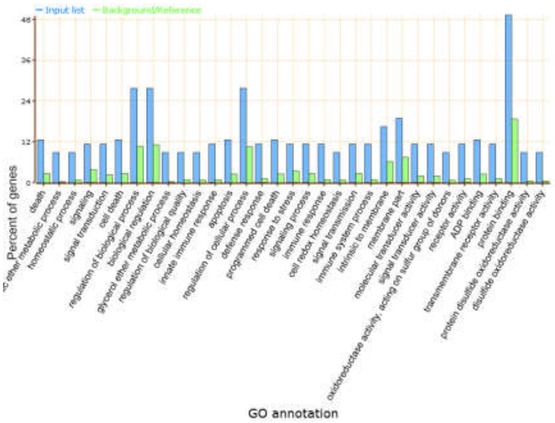

P. alba (Chr01, 1175321bp)

P. devidiana (Chr19, 1441218 bp)

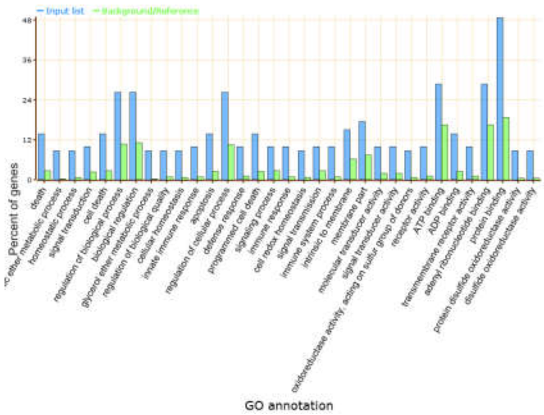

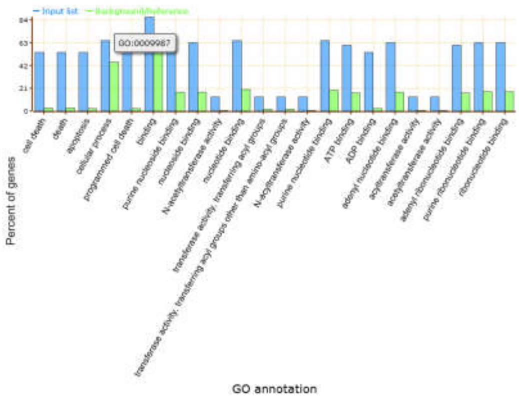

P. cathayana (Chr19, 1450930 bp)

P. simonii (Chr01,1706425 bp)

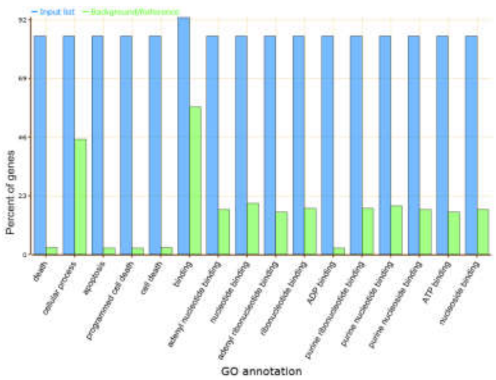

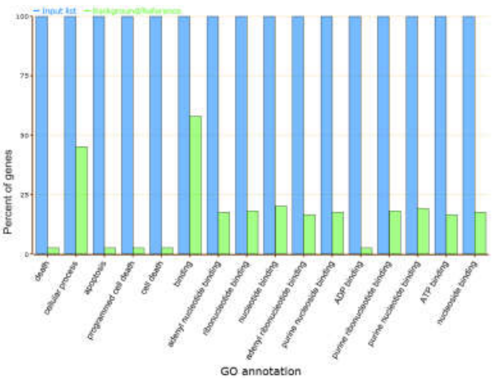

P. ussuriensis (Chr01, 808562 bp)

P. maximowiczii (Chr01, 333616 bp)

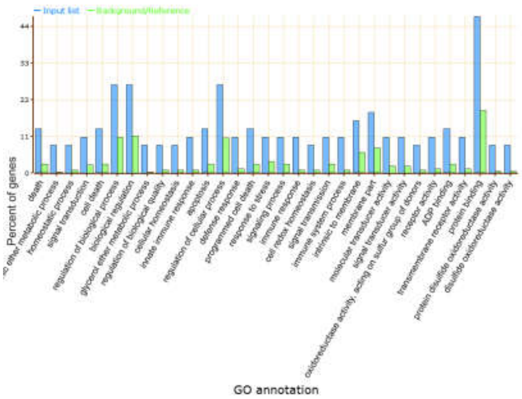

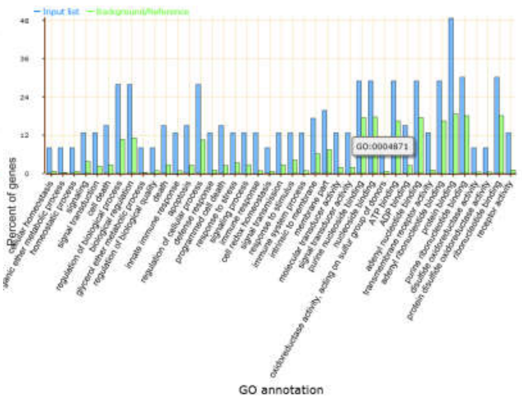

P. nigra (Chr19, 1465273 bp) *P. deltoids*(Chr19,1488370 bp)

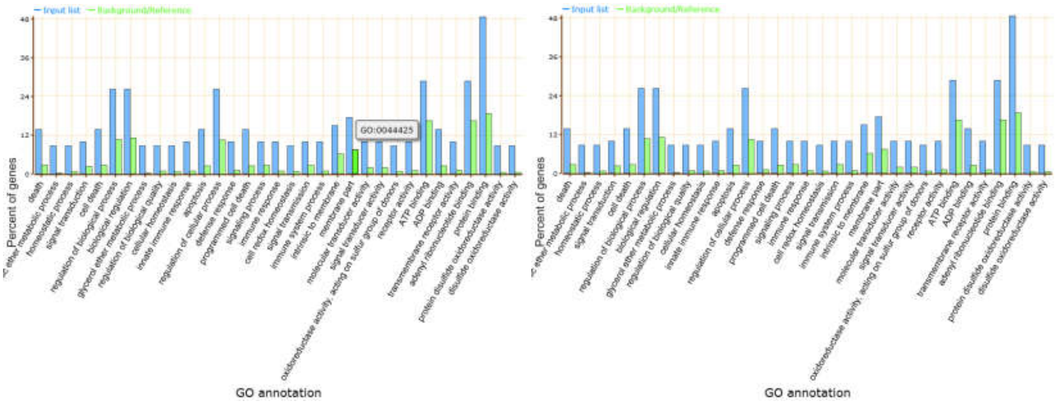

P. lasiocarpa (Chr19, 1450927 bp) *P. euphratica* (Chr19,1450918 bp)

Supplementary Figure 7 Significantly enriched GO terms of genes located in one of the large deletions of the 10 poplar species. The chromosome and size of the large deletion in each species were presented under the panel.

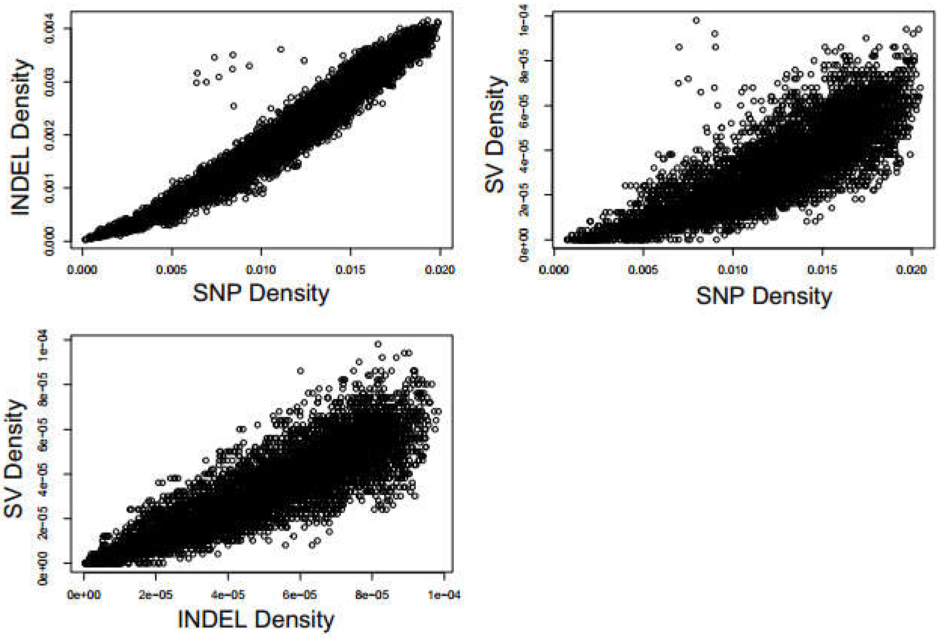

Supplementary Figure 8 Correlation plot for SNP, indel, and SV density (excluding CTXs and ITXs from the SV density calculation) in the *P. alba* genome (window: 500 kb, step: 50 kb).

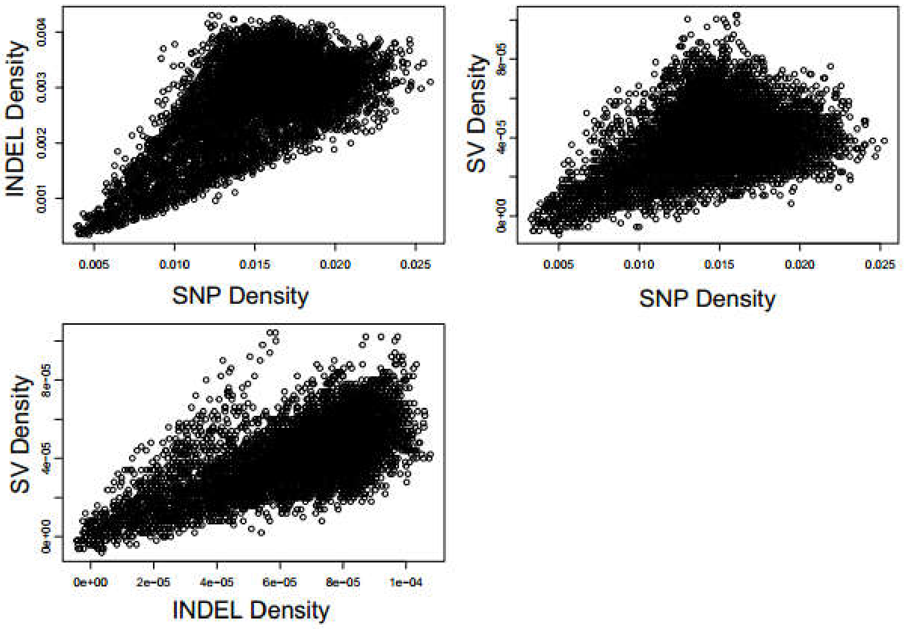

Supplementary Figure 9 Correlation plot for SNP, indel, and SV density (excluding CTXs and ITXs from the SV density calculation) in the *P. cathayana* (right) genome (window: 500 kb, step: 50 kb).

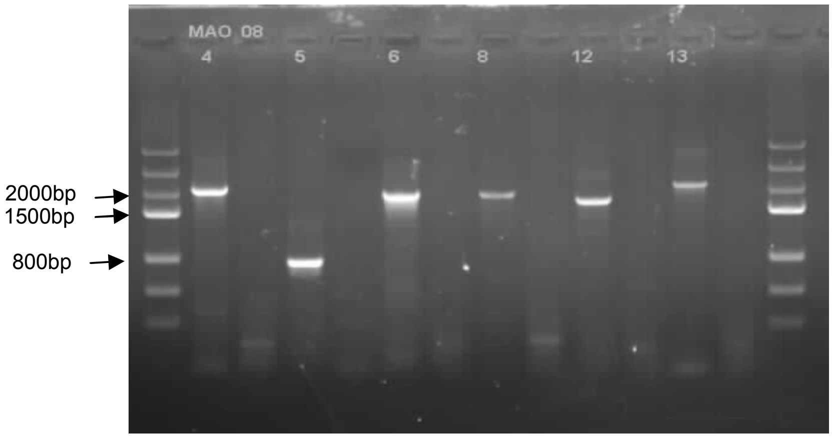

Supplementary Figure 10 Validation of bioinformatic-detected deletions in *P. lasiocarpa* based on PCR. For each deletion, the primers within the deletion region were designed according to the reference genome sequence. Lanes 1 and 14 are DNA markers; lanes 2, 4, 6, 8, and 10 are the PCR results using *P. trichocarpa* genomic DNA as templates; and lanes 3, 5, 7, 9, and 11 are the PCR results using *P. lasiocarpa* genomic DNA as templates. The Arabic numerals (4, 5, 6, 8, 12, and 13) represent the PCR primer pairs (e.g., 4 represents the primers of 08DEL-L4, 08DEL-R4). DNA marker III (Biosharp Inc., Seoul, South Korea) was used for the markers.

Supplementary Tables

Supplementary Table 1 Summary of the 10 sequenced poplar species and collection locations.

clone	Species	Section	**Geographic distribution**
01	*P. alba* L.	Leuce Duby, subsect. Albidae Dode.	87°40', 47°26'
17	*P. devidiana* Dode.	Leuce Duby, subsect. Trepidae Dode.	126°31', 43°47'
06	*P. cathayana* Rehd.	Tacamahaca Spach.	116°12', 39°57'
07	*P. simonii* Carr.	Tacamahaca Spach.	116°12’ , 39°59'
09	*P. ussuriensis* Kom.	Tacamahaca Spach.	129°1', 47°0'
19	*P. maximowiczii* Henry.	Tacamahaca Spach.	124°47', 40°44'
11	*P. nigra* L.	Aigeiros Duby.	87°36', 47°4'
12	*P. deltoides* Bartr.	Aigeiros Duby.	119°10', 39°43'
08	*P. lasiocarpa* Oliv.	Leucoides Spach.	110°56', 31°4'
15	*P. euphratica* Oliv.	Turanga Bge.	86°22', 41°17'

Supplementary Table 2 Resequencing summary of the 10 poplar species.

Sections/ species	GC Rate (%)	Quality value >=20 Rate (%)	Quality value>=30 Rate (%)	Production		Insert Size (bp)	Coverage rate (%)	Mapped Rate (%)	UniqMapped Rate (%)	Sequencing depth(X)
				Reads (M)	Bases (G)					
**Sect. Leuce Duby**										
P. alba L.	37.76	96.44	90.28	182.29	16.41	482	58.90	59.95	31.32	34.08
P. devidiana Dode.	37.99	97.13	91.74	182.77	16.45	469	59.05	59.34	31.53	35.42
**Sect. Tacamahaca Spach.**										
P. cathayana Rehd.	36.71	96.73	90.81	182.89	16.46	473	86.94	77.39	44.85	35.13
P. simonii Carr.	36.92	96.35	89.93	182.50	16.43	469	79.83	76.74	44.51	35.55
P. ussuriensis Kom.	36.63	95.42	87.92	181.53	16.34	471	85.41	81.04	49.39	35.42
P. maximowiczii Henry.	35.40	96.59	90.98	182.98	16.47	470	85.44	79.80	47.74	36.15
**Sect. Aigeiros Duby.**										
P. nigra L.	36.90	97.14	91.76	182.73	16.45	468	76.54	75.25	43.23	35.02
P. deltoides Bartr.	35.95	92.47	83.19	166.74	15.01	473	79.10	72.73	41.03	32.57
**Sect. Leucoides Spach.**										
P. lasiocarpa Oliv.	37.64	96.36	90.29	182.71	16.44	480	80.18	77.20	43.13	35.35
**Sect. Turanga Bge.**										
P. euphratica Oliv.	34.99	90.02	89.15	182.68	16.44	470	54.16	59.48	27.26	34.15
Total				1809.83	162.89					

Note: Coverage rate (%)" represents the percentage of the genome covered.

Supplementary Table 3 Summary of all detected SNPs from the 10 poplar species and their distributions in different genic regions.

Sections/ species	SNP	Homo	Hete	CDS	Syn_CDS	Nonsyn_CDS	stop_ codon	UTR	Intron	IG
Sect. Leuce Duby										
P. alba L.	5,176,530	3,826,237	1,350,293	866,114	416,756	449,358	5,074	506,466	1,707,434	2,096,516
P. devidiana Dode.	5,424,245	3,735,247	1,688,998	900,957	431,136	469,821	5,382	518,489	1,739,881	2,264,918
Sect. Tacamahaca Spach.										
P. cathayana Rehd.	6,564,546	4,601,872	1,962,674	436,754	194,544	242,210	3,759	264,518	954,800	4,908,474
P. simonii Carr.	6,337,615	4,967,333	1,370,282	505,688	227,631	278,057	4,230	308,226	1,103,421	4,420,280
P. ussuriensis Kom.	6,741,894	4,496,649	2,245,245	430,110	191,063	239,047	3,749	261,239	945,413	5,105,132
P. maximowiczii Henry.	6,936,296	4,809,778	2,126,518	427,390	190,731	236,659	3,657	261,391	952,832	5,294,683
Sect. Aigeiros Duby.										
P. nigra L.	6,098,042	5,132,380	965,662	508,475	234,138	274,337	3,913	320,278	1,130,870	4,138,419
P. deltoides Bartr.	6,398,415	4,753,823	1,644,592	516,748	236,906	279,842	4,183	332,515	1,159,007	4,390,145
Sect. Leucoides Spach.										
P. lasiocarpa Oliv.	5,870,412	4,782,476	1,087,936	460,136	204,935	255,201	3,734	281,338	975,999	4,152,939
Sect. Turanga Bge.										
P. euphratica Oliv.	5,067,560	4,258,535	809,025	874,009	421,347	452,662	5,175	530,449	1,848,226	1,814,876
Total	60,615,555	45,364,330	15,251,225	5,926,381	2,749,187	3,177,194	42,856	3,584,909	12,517,883	38,586,382

Note: Homo: homozygous; hete: heterozygous; syn: synonymous; Nonsyn: nonsynonymous; UTR: untranslated region; IG: intergenic region

Supplementary Table 4 Heterozygous/homozygous SNP ratio in the 10 poplar species.

**Sections/ species**	**SNP**	**Homo**	**Hete**	**Hete/Homo**
Sect. Leuce Duby				
P. alba L.	5,176,530	3,826,237	1,350,293	0.35
P. devidiana Dode.	5,424,245	3,735,247	1,688,998	0.45
Sect. Tacamahaca Spach.				
P. cathayana Rehd.	6,564,546	4,601,872	1,962,674	0.43
P. simonii Carr.	6,337,615	4,967,333	1,370,282	0.28
P. ussuriensis Kom.	6,741,894	4,496,649	2,245,245	0.50
P. maximowiczii Henry.	6,936,296	4,809,778	2,126,518	0.44
Sect. Aigeiros Duby.				
P. nigra L.	6,098,042	5,132,380	965,662	0.19
P. deltoides Bartr.	6,398,415	4,753,823	1,644,592	0.35
Sect. Leucoides Spach.				
P. lasiocarpa Oliv.	5,870,412	4,782,476	1,087,936	0.23
Sect. Turanga Bge.				
P. euphratica Oliv.	5,067,560	4,258,535	809,025	0.19

Note: Homo: homozygous; Hete: heterozygous.

Supplementary Table 5 Nonsynonymous/synonymous substitutions ratio in the 10 poplar species.

**Sections/ species**	**SNP**	**Syn_CDS**	**Nonsyn_CDS**	**Nonsyn/Syn**
Sect. Leuce Duby				
P. alba L.	5,176,530	416,756	449,358	1.08
P. devidiana Dode.	5,424,245	431,136	469,821	1.09
Sect. Tacamahaca Spach.				
P. cathayana Rehd.	6,564,546	194,544	242,210	1.25
P. simonii Carr.	6,337,615	227,631	278,057	1.22
P. ussuriensis Kom.	6,741,894	191,063	239,047	1.25
P. maximowiczii Henry.	6,936,296	190,731	236,659	1.24
Sect. Aigeiros Duby.				
P. nigra L.	6,098,042	234,138	274,337	1.17
P. deltoides Bartr.	6,398,415	236,906	279,842	1.18
Sect. Leucoides Spach.				
P. lasiocarpa Oliv.	5,870,412	204,935	255,201	1.25
Sect. Turanga Bge.				
P. euphratica Oliv.	5,067,560	421,347	452,662	1.07
Average				1.16

Note: syn, synonymous; Nonsyn: nonsynonymous;

Supplementary Table 6 Classification of nucleotide substitutions in SNPs detected in the 10 poplar species.

Sections/ species	**Transitions(Ts)**		**Transversions(Tv)**				**Ts/Tv ratio**
	**C / T**	**A / G**	**A / T**	**A / C**	**T / G**	**C / G**	
Sect. Leuce Duby							
P. alba L.	1,525,757	1,527,967	735,653	532,282	531,184	323,687	1.44
P. devidiana Dode.	1,586,863	1,592,096	775,893	561,649	562,126	345,618	1.42
Sect. Tacamahaca Spach.							
P. cathayana Rehd.	2,133,125	2,136,906	805,273	581,400	582,042	325,800	1.86
P. simonii Carr.	2,015,570	2,015,992	803,975	584,471	585,986	331,621	1.75
P. ussuriensis Kom.	2,192,089	2,197,789	830,027	594,694	595,986	331,309	1.87
P. maximowiczii Henry.	2,253,248	2,260,210	864,186	609,377	611,445	337,830	1.86
Sect. Aigeiros Duby.							
P. nigra L.	194,1973	1,944,416	764,572	561,669	563,242	322,170	1.76
P. deltoides Bartr.	2,027,974	2,030,171	823,081	587,973	589,704	339,512	1.73
Sect. Leucoides Spach.							
P. lasiocarpa Oliv.	1,885,256	1,888,632	722,704	535,152	535,793	302,875	1.80
Sect. Turanga Bge.							
P. euphratica Oliv.	1,471,287	1,472,169	721,619	533,713	533,397	335,375	1.39

Supplementary Table 7 Significantly enriched GO terms of genes with common SNPs of the 10 poplar species.

GO term	Ontology	Description	Number in input list	Number in BG/Ref	p-value	FDR
GO:0019219	P	regulation of nucleobase, nucleoside, nucleotide and nucleic acid metabolic process	888	1568	0.00008	0.042
GO:0006807	P	nitrogen compound metabolic process	1636	3018	5.70E-05	0.042
GO:0051171	P	regulation of nitrogen compound metabolic process	888	1568	8.00E-05	0.042
GO:0045449	P	regulation of transcription	885	1562	7.90E-05	0.042
GO:0006355	P	regulation of transcription, DNA-dependent	884	1560	7.90E-05	0.042
GO:0006139	P	nucleobase, nucleoside, nucleotide and nucleic acid metabolic process	1453	2651	3.90E-05	0.042
GO:0016070	P	RNA metabolic process	1136	2029	3.00E-05	0.042
GO:0051252	P	regulation of RNA metabolic process	884	1560	7.90E-05	0.042
GO:0010556	P	regulation of macromolecule biosynthetic process	889	1577	0.00012	0.044
GO:0009889	P	regulation of biosynthetic process	889	1577	0.00012	0.044
GO:0080090	P	regulation of primary metabolic process	893	1585	0.00012	0.044
GO:0031326	P	regulation of cellular biosynthetic process	889	1577	0.00012	0.044
GO:0030528	F	transcription regulator activity	661	1110	1.20E-05	0.04
GO:0003677	F	DNA binding	1211	2173	2.80E-05	0.046

Note: F:Mollecular Function; P:Biological Process

Supplementary Table 8 Distribution of large-effect SNPs in the genic regions of the 10 poplar species.

**Sections/ species**	**SNPs**	**Premature stop SNPs**	**Stop to non-stop codon SNPs**	**Start to non-start codon SNPs**	**Total large effect SNPs**
Sect. Leuce Duby					
P. alba L.	5,176,530	5,074	1,070	14,769	20,913 (0.40%)[a]
P. devidiana Dode.	5,424,245	5,382	1,106	15,496	21,984 (0.41%)
Sect. Tacamahaca Spach.					
P. cathayana Rehd.	6,564,546	3,759	776	7,781	12,316 (0.19%)
P. simonii Carr.	6,337,615	4,230	846	9,008	17,468 (0.22%)
P. ussuriensis Kom.	6,741,894	3,749	761	7,707	12,217 (0.18%)
P. maximowiczii Henry.	6,936,296	3,657	755	7,772	12,184 (0.18%)
Sect. Aigeiros Duby.					
P. nigra L.	6,098,042	3,913	838	8,997	13,748 (0.23%)
P. deltoides Bartr.	6,398,415	4,183	833	9,179	14,195 (0.22%)
Sect. Leucoides Spach.					
P. lasiocarpa Oliv.	5,870,412	3,734	805	8,321	12,860 (0.22%)
Sect. Turanga Bge.					
P. euphratica Oliv.	5,067,560	5,175	1,068	14,722	20,965 (0.41%)
Total	6,0615,555	42,856	12,242	103,752	155,466
Percentage of the total		0.071	0.015	0.171	0.256

a.The percentage of the large effect SNPs in the total SNP in each species.

Supplementary Table 9 Summery of all detected small indels from the 10 poplar species and their distributions in different genic regions.

**Sections/ speices**	**Indel**	**Insertion**	**Deletion**	**UTR**	**CDS**	**frameshift**	**Non-frameshift**	**D/Iratio**	**Shift/Non_shift**
Sect. Leuce Duby									
P. alba L.	849,526	410,724	438,802	107,974	33,395	19,493	13,902	1.07	1.40
P. devidiana Dode.	923,668	439,617	484,051	114,827	36,425	21,241	15,184	1.10	1.40
Sect. Tacamahaca Spach.									
P. cathayana Rehd.	1,140,033	572,155	567,878	64,319	22,094	13,102	8,992	0.99	1.46
P. simonii Carr.	1,095,352	542,563	552,789	73,991	24,777	14,812	9,965	1.02	1.49
P. ussuriensis Kom.	1,178,680	601,362	577,318	62,926	21,643	12,812	8,831	0.96	1.45
P. maximowiczii Henry.	1,244,563	632,081	612,482	62,846	21,510	12,652	8,858	0.97	1.43
Sect. Aigeiros Duby.									
P. nigra L.	1,027,498	511,937	515,561	73,515	23,334	13,752	9,582	1.01	1.44
P. deltoides Bartr.	1,091,146	543,963	547,183	74,608	23,525	13,934	9,591	1.01	1.45
Sect. Leucoides Spach.									
P. lasiocarpa Oliv.	966,135	491,263	474,872	66,464	22,592	13,217	9,375	0.97	1.41
Sect. Turanga Bge.									
P. euphratica Oliv.	801,186	367,867	433,319	105,016	31,118	18,479	12,639	1.18	1.46
Total	10,317,787	5,113,532	5,204,255	806,486	260,413	153,494	106,919		

Note: D/I ratio: deletions/insertions; Shift/non_shift: fameshift indels/nonframeshift indels

Supplementary Table 10 List of primers used for the SNP and indel validation in *P. alba* based on PCR and Sanger sequencing.

primer	primer sequence	Product size(bp)	Chr.	Position
SN2	AGACTCGGCGGAATCATGT<br>GACATGTCGTGGGTGTCAAC	472	Chr02	7183616..7184116
SN4	GGGACACATGAAGTGAATTGG<br>TTTCTTTGAGTTCTCCGCAAG	383	Chr03	10567544..10567944
SN5	GACCCTCACGCCTAGAACAA<br>GGGCTCTTGCTACTCCACTC	445	Chr04	17226103..17226602
SN8	AGTGGCTGCATCTGCAAGTA<br>CAAGTCTCCAAATGTCAAGCTG	425	Chr06	24710499..24710998
S1	AAGAGGCACCCATCTTAGCT<br>GCCTTAAATGGGATGCTGCA	420	Chr19	15289305..15289805
S3	AGTTCAAGCCCGATCAGTCA<br>GGAACGTAAACCATGCTCATAGT	478	Chr01	38985248..38985748
S4	GTCCAAGAGCAGCCCTGA<br>GAAAGCAGAAAGTCCATGGCA	401	Chr07	1939137..1939637
ID-3	GCGAATGAATAGGCCAGAGA<br>AGGTATGGCTTCTGCAAGGA	413	Chr04	2488007..2488607
ID-5	TGATGAAGCAGACACCGAGT<br>GTTGACATGACCTGCGTGAG	402	Chr13	9861266..9861866
ID-6	GCTAGCTCTCCCTCCCAGAG<br>CAAGCTTGGATGTCTATGTTGC	535	Chr02	4553634.. 4554234
ID-7	TCCCATCTCCTTGAGCTTGT<br>TTAAATGCCCATGCCTTTGT	407	Chr11	7828319..7828919
ID-8	GTCGGAACCAATTCCCCTAT<br>CCAGGCTCACCACATATCAA	498	Chr10	1868548..1868608
ID-11	TAATGCAACGCCTGTTCATC<br>CCTTCATTTACCGCCAGAAA	494	Chr15	9547830..9548430
ID-12	GCTGAATCGCCAGCAGTAAT<br>CTGTCCGAGGATGGAAGTGT	531	Chr09	6326406.. 6327004

Supplementary Table 11 List of primers used for the SNP and indel validation in *P. deltoides* based on PCR and Sanger sequencing.

primer	primer sequence	Product size(bp)	Chr.	Position
ID -1	TGCTCGTCGATCATTGTTTTCT<br>AGCCCAACTCATCACCAGAA	909	Chr02	9342957..9344000
ID -3	TCATCATATAGTGGACAATGGGC<br>TGGGATGTGATAATCAAAGGTGC	900	Chr10	318326..319370
ID -4	TGGGAAGAAAGGAGTTGGCG<br>ACGGCAAGCTGACTCCATAA	906	Chr10	1335978..13360822
ID -5	ACCGGAGCAATATCAACAAGGA<br>CTTCAAGGTGAGGGCAGGTG	628	Chr07	10042968..10044010
ID -12	TCCGTACACTTTGCCATTTCA<br>TTTCCACGCACAGCTTATGG	1063	Chr05	4781754..4782870
ID -14	TCCAAATTCCAATCATGACACGT<br>CGCCTCTTACTGGTGGGAAT	1021	Chr13	4488053..4489104

Supplementary Table 12 List of primers used for the PCR-based verification of several predicted deletions in *P. lasiocarpa*.

Chromosome	Primer name	Primer sequence	Start (*P. trichocarpa*)	End (*P. trichocarpa*)	Deletion length(bp)	*P. trichocarpa* size(bp)	*P. lasiocarpa* size(bp)
Chr09	08DEL-L4<br>08DEL-R4	GACCCTACATTCACGAGTCTTTT<br>TCCTCCTCCGTAACTTTGAACA	594,367	596,635	2,314	2,176	0
Chr01	08DEL-L5<br>08DEL-R5	GTGAAACGCTGAGCAGTCTT<br>ACCCGTGAAATCAGGTTAACC	22,573,650	22,575,928	2,279	794	0
Chr18	08DEL-L6<br>08DEL-R6	GGGCTTTTAATGGGCTTCGTAA<br>TTTCCCTTTCATACAGCCGC	2,080,068	2,082,396	2,337	1,893	0
Chr10	08DEL-L8<br>08DEL-R8	GTGGATCGCTTGGGAGGT<br>TTGATGCCCATATGACTTGAAAG	16,891,783	16,894,075	2,304	1,993	0
Chr03	08DEL-L12<br>08DEL-R12	TTTGGTCAAATCAGGGGCTT<br>TGTGGCAAATGTGTGGAGAC	3,503,829	3,506,117	2,311	1,791	0
Chr18	08DEL-L13<br>08DEL-R13	CGACAAGAGGAGGGAGAAAGT<br>TGAGCCAAGTCAAGGTCCC	5,934,833	5,937,375	2,588	2,258	0

Supplementary Table 13 Ks range of the 10 poplar species.

**Sections/ species**	**Ks range**
Sect. Leuce Duby	
P. alba L.	0.03-0.04
P. devidiana Dode.	0.03-0.04
Sect. Tacamahaca Spach.	
P. cathayana Rehd.	0.01-0.02
P. simonii Carr.	0.01-0.02
P. ussuriensis Kom.	0.01-0.02
P. maximowiczii Henry.	0.01-0.02
Sect. Aigeiros Duby.	
P. nigra L.	0.01-0.02
P. deltoides Bartr.	0.01-0.02
Sect. Leucoides Spach.	
P. lasiocarpa Oliv.	0.01-0.02
Sect. Turanga Bge.	
P. euphratica Oliv.	0.04-0.05
