# Supplementary material for: The poplar pangenome provides insights into the evolutionary history of the genus
Source: Commun Biol. 2019 Jun 18;2:215. doi: 10.1038/s42003-019-0474-7 (PMC6581948; doi:10.1038/s42003-019-0474-7)
Supplement: Supplementary file 2 — Description of Additional Supplementary Files [file 42003_2019_474_MOESM2_ESM.docx]

**Description of additional supplementary items**

Supplementary Data 1 SNPs shared among the 10 poplar species.

Supplementary Data 2 Significantly enriched GO terms of genes with non-start codon SNPs in the 10 poplar species.

Supplementary Data 3 Significantly enriched GO terms of genes with stop codons SNPs in the 10 poplar species.

Supplementary Data 4 Small indels shared among the 10 poplar species.

Supplementary Data 5 Significantly enriched GO terms of genes with frameshift indels in the 10 poplar species.

Supplementary Data 6 Significantly enriched GO terms of heterozygous non-start codon SNPs genes in the 10 poplar species.

Supplementary Data 7 Significantly enriched GO terms of heterozygous stop codon SNPs genes in the 10 poplar species.

Supplementary Data 8 Significantly enriched GO terms of genes with homozygous large-effect SNPs in the 10 poplar species.

Supplementary Data 9 Significantly enriched GO terms of heterozygous frameshift indel genes in the 10 poplar species.

Supplementary Data 10 Significantly enriched GO terms of genes with homozygous frameshift Indels in the 10 poplar species.

Supplementary Data 11 The reproduction-related genes significantly enriched in in genes with loss-of-function mutations in six poplar species.
