# Supplementary material for: The poplar pangenome provides insights into the evolutionary history of the genus
Source: Commun Biol. 2019 Jun 18;2:215. doi: 10.1038/s42003-019-0474-7 (PMC6581948; doi:10.1038/s42003-019-0474-7)
Supplement: Supplementary file 14 — Reporting Summary [file 42003_2019_474_MOESM14_ESM.pdf]

nature research

Corresponding author(s): Xiaohua Su, Bingyu Zhang

Last updated by author(s): May 23, 2019

Reporting Summary

Nature Research wishes to improve the reproducibility of the work that we publish. This form provides structure for consistency and transparency in reporting. For further information on Nature Research policies, see Authors & Referees and the Editorial Policy Checklist.

Statistics

For all statistical analyses, confirm that the following items are present in the figure legend, table legend, main text, or Methods section.

n/a	Confirmed	
☐	☒	The exact sample size (n) for each experimental group/condition, given as a discrete number and unit of measurement
☐	☒	A statement on whether measurements were taken from distinct samples or whether the same sample was measured repeatedly
☐	☒	The statistical test(s) used AND whether they are one- or two-sided<br>*Only common tests should be described solely by name; describe more complex techniques in the Methods section.*
☐	☒	A description of all covariates tested
☐	☒	A description of any assumptions or corrections, such as tests of normality and adjustment for multiple comparisons
☐	☒	A full description of the statistical parameters including central tendency (e.g. means) or other basic estimates (e.g. regression coefficient) AND variation (e.g. standard deviation) or associated estimates of uncertainty (e.g. confidence intervals)
☐	☒	For null hypothesis testing, the test statistic (e.g. F, t, r) with confidence intervals, effect sizes, degrees of freedom and P value noted<br>*Give P values as exact values whenever suitable.*
☒	☐	For Bayesian analysis, information on the choice of priors and Markov chain Monte Carlo settings
☒	☐	For hierarchical and complex designs, identification of the appropriate level for tests and full reporting of outcomes
☐	☒	Estimates of effect sizes (e.g. Cohen's d, Pearson's r), indicating how they were calculated

Our web collection on statistics for biologists contains articles on many of the points above.

Software and code

Policy information about availability of computer code

Data collection	We used P. trichocarpa genome v.3.0 (ftp://ftp.jgi-psf.org/pub/compgen/phytozome/v9.0/Ptrichocarpa/) as the reference. The softwares used in this study were not commercial software.
Data analysis	The data analysis in this study didn't used commercial software.

For manuscripts utilizing custom algorithms or software that are central to the research but not yet described in published literature, software must be made available to editors/reviewers. We strongly encourage code deposition in a community repository (e.g. GitHub). See the Nature Research guidelines for submitting code & software for further information.

Data

Policy information about availability of data

All manuscripts must include a data availability statement. This statement should provide the following information, where applicable:

- Accession codes, unique identifiers, or web links for publicly available datasets
- A list of figures that have associated raw data
- A description of any restrictions on data availability

The data of this study is available in SRA (PRJNA540895).

Field-specific reporting

Please select the one below that is the best fit for your research. If you are not sure, read the appropriate sections before making your selection.

☒ Life sciences ☐ Behavioural & social sciences ☐ Ecological, evolutionary & environmental sciences

October 2018

Life sciences study design

All studies must disclose on these points even when the disclosure is negative.

Sample size	We resequenced 10 clones from 10 species (i.e., one clone per species) of genus Populus. the clones were collected in their wild distribution regions, and the clones had the species-specific morphological characteristics.
Data exclusions	No data were excluded from the analyses.
Replication	We used PCR and Sanger sequencing to validated some of the SNPs and indels randomly selected from two samples. We used PCR amplification to validate the SVs randomly selected from the SV of one sample.
Randomization	The clone from each species was randomly selected from the clones with species-specific morphological characteristics. And the SNPs, indels and SVs used for validation were also randomly selected.
Blinding	The investigators were blinded to group allocation during data collection and analysis.

Reporting for specific materials, systems and methods

We require information from authors about some types of materials, experimental systems and methods used in many studies. Here, indicate whether each material, system or method listed is relevant to your study. If you are not sure if a list item applies to your research, read the appropriate section before selecting a response.

Materials & experimental systems

n/a	Involved in the study	
☒	☐	Antibodies
☒	☐	Eukaryotic cell lines
☒	☐	Palaeontology
☐	☒	Animals and other organisms
☒	☐	Human research participants
☒	☐	Clinical data

Methods

n/a	Involved in the study	
☒	☐	ChIP-seq
☒	☐	Flow cytometry
☒	☐	MRI-based neuroimaging

Animals and other organisms

Policy information about studies involving animals; ARRIVE guidelines recommended for reporting animal research

Laboratory animals	The study did not involve laboratory animals.
Wild animals	The study did not involve wild animals.
Field-collected samples	The 10 species were collected from their natural distribution areas and then planted in a greenhouse under a controlled temperature (24~30℃℃℃℃ Celsius degree) and natural sunlight.in Beijing, China.
Ethics oversight	No ethical approval was required because all the species used in this study were not in dangerous species.

Note that full information on the approval of the study protocol must also be provided in the manuscript.
